# Functions and Regulation of Translation Elongation Factors

**DOI:** 10.3389/fmolb.2021.816398

**Published:** 2022-01-19

**Authors:** Benjin Xu, Ling Liu, Guangtao Song

**Affiliations:** ^1^ Department of Medical Laboratory Science, Fenyang College, Shanxi Medical University, Fenyang, China; ^2^ Institute of Biophysics, Chinese Academy of Sciences, Beijing, China

**Keywords:** translation, elongation factors, regulation, expression, tumorigenesis

## Abstract

Translation elongation is a key step of protein synthesis, during which the nascent polypeptide chain extends by one amino acid residue during one elongation cycle. More and more data revealed that the elongation is a key regulatory node for translational control in health and disease. During elongation, elongation factor Tu (EF-Tu, eEF1A in eukaryotes) is used to deliver aminoacyl-tRNA (aa-tRNA) to the A-site of the ribosome, and elongation factor G (EF-G, EF2 in eukaryotes and archaea) is used to facilitate the translocation of the tRNA_2_-mRNA complex on the ribosome. Other elongation factors, such as EF-Ts/eEF1B, EF-P/eIF5A, EF4, eEF3, SelB/EFsec, TetO/Tet(M), RelA and BipA, have been found to affect the overall rate of elongation. Here, we made a systematic review on the canonical and non-canonical functions and regulation of these elongation factors. In particular, we discussed the close link between translational factors and human diseases, and clarified how post-translational modifications control the activity of translational factors in tumors.

## Introduction

**FIGURE 1:**
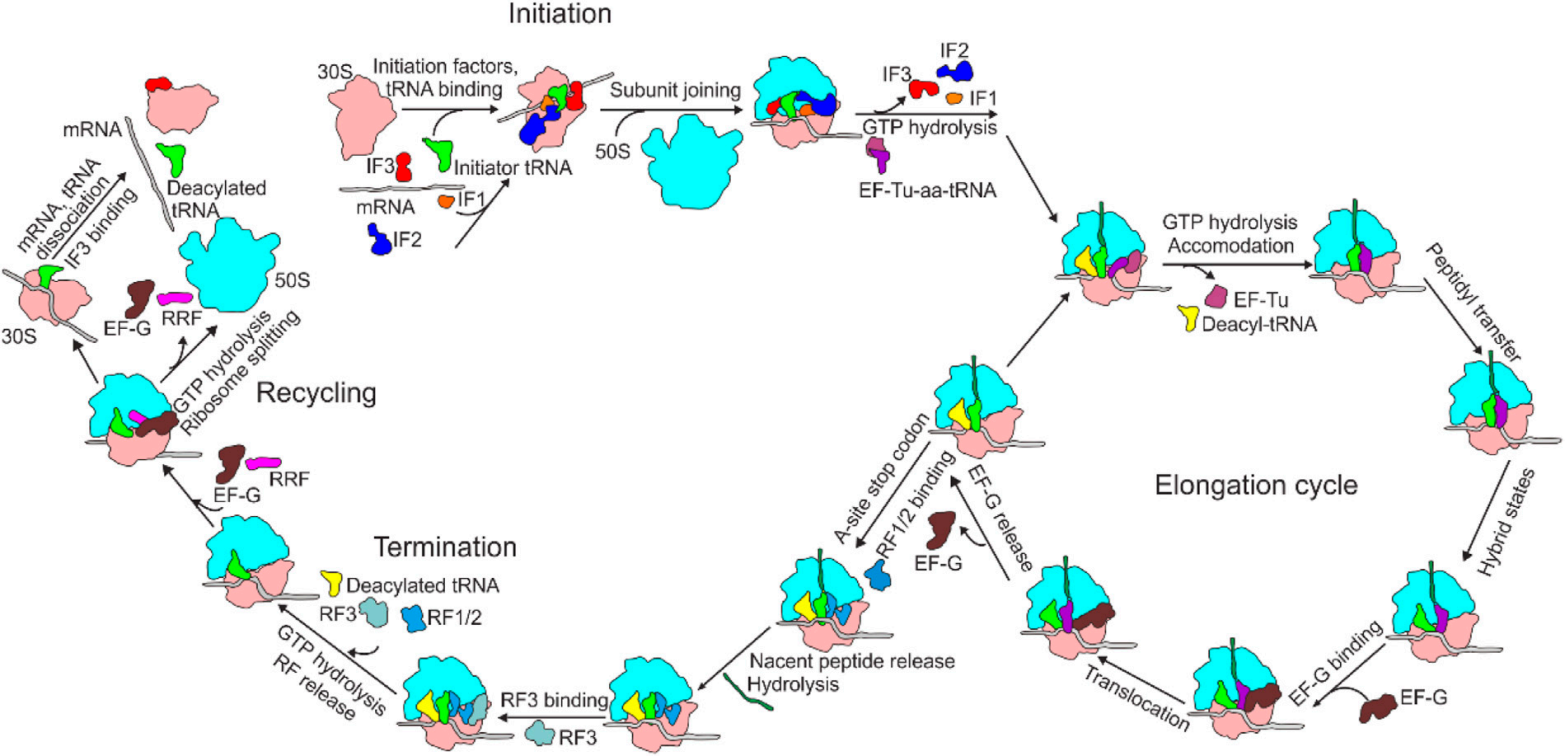
Overview of bacterial translation cycle. aa-tRNA, aminoacyl-tRNA; EF, elongation factor; IF, initiation factor; RF, release factor; RRF, ribosome recycling factor.

With the development of structural biology, especially the rapid development of cryo-electron microscopy (cryo-EM) (Benjin and Ling, 2019), the mechanism of intracellular protein translation and its regulation have been gradually clarified. As described by the central dogma, translation is the final stage of gene expression, during which the genetic information carried by an mRNA is transformed into the amino acid sequence of a protein catalyzed by a ribosome (Voorhees and Ramakrishnan, 2013). Translation is a highly dynamic and cyclic process, which is composed of four steps: initiation, elongation, termination, and ribosome recycling (Figure 1). During translation initiation, the ribosome, with an initiator fMet-tRNA^fMet^ in the P-site, is assembled on mRNA with the assistance of the three initiation factors (IF1-3). The ribosome complex is then ready to accept the first elongator tRNA and form the first peptide bond, which marks the beginning of the next stage, elongation. During elongation, an aa-tRNA enters the ribosome A-site with the help of EF-Tu. If proper base-pairing between the three bases of the mRNA codon and those of the aa-tRNA anticodon is established, the aa-tRNA is cognate, a peptide bond is formed with the peptide attached to the tRNA in the P-site. The peptidyl-tRNA is then moved from the A-to the P-site, and the deacylated tRNA in the P-site is moved to the E-site. The mRNA is coordinately translocated by one codon. Termination occurs when the ribosome reaches a terminator codon in an mRNA. The nascent peptide is hydrolyzed by the release factors (RF1/2) and dissociated from the ribosome. In the end, the ribosome is split into two subunits by the concerted action of EF-G and RRF, releasing the deacylated tRNA and mRNA, and preparing for a new round of translation initiation. This step is called ribosome recycling.

**FIGURE 2:**
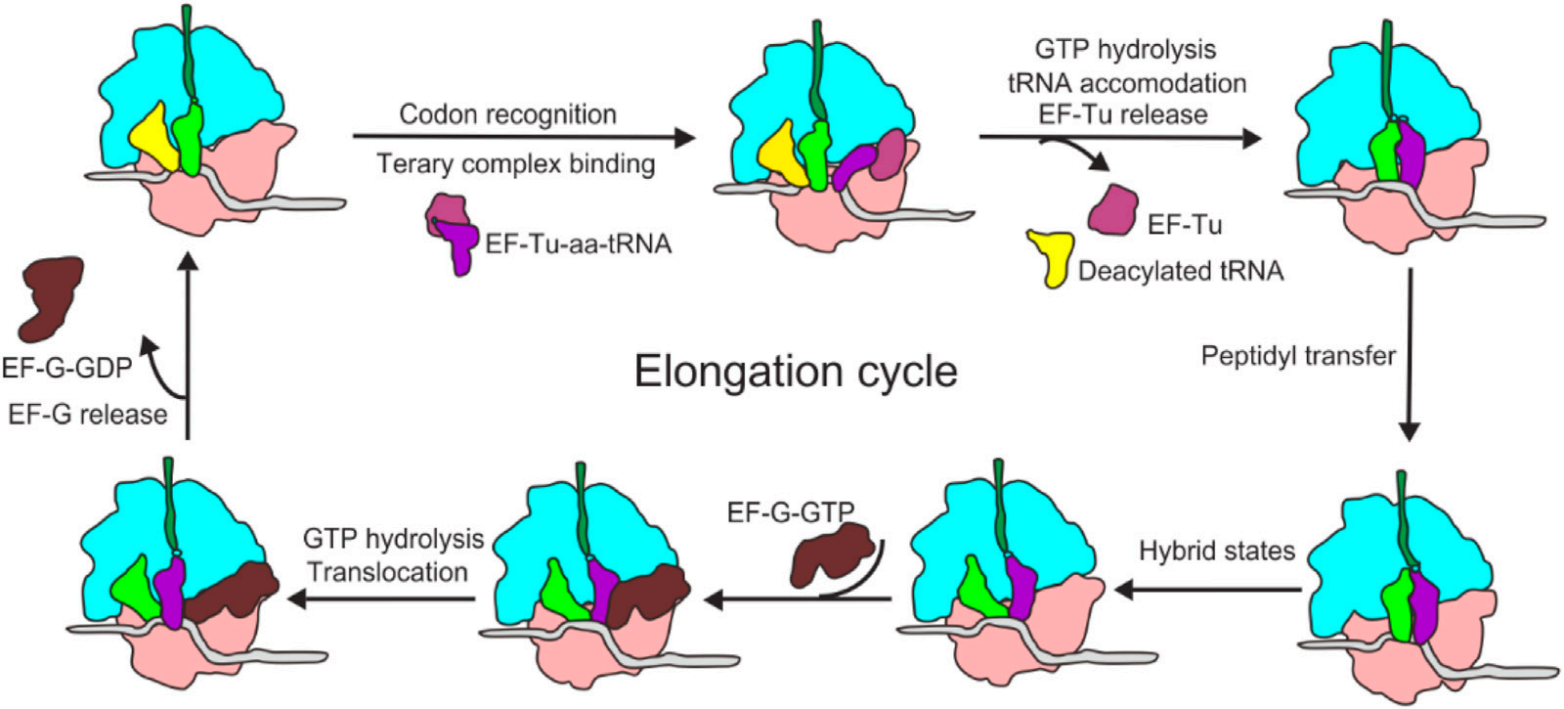
Schematic of the bacterial elongation cycle. EF-Tu delivers aa-tRNA to the A-site of the ribosome, where the ribosome decodes for the cognate tRNA. After aa-tRNA is fully accommodated and peptide bond formation, EF-G facilitates translocation of the tRNA_2_-mRNA duplex, then the next round of elongation begins.

Translation elongation is a process of repeated decoding, peptidyl transfer and tRNA_2_-mRNA translocation. It starts with the binding of the second aminoacyl-tRNA at the A-site. During elongation, an aa-tRNA is delivered to the ribosome as a ternary complex (TC) with elongation factor Tu (EF-Tu) and GTP (EF-Tu∙GTP∙aa-tRNA). Binding of cognate aa-tRNA to mRNA in the A site of the ribosome induces the crucial and generally conserved bases A1493, A1492 and G530 to flip out and interact with the minor groove of the mRNA-tRNA duplex (Schmeing and Ramakrishnan, 2009), which further induces a domain closure in the 30S subunit (Ogle et al., 2002). 30S domain closure makes the 30S shoulder towards the ternary complex (Ogle and Ramakrishnan, 2005), leading to the stimulation of the GTP hydrolysis by EF-Tu and acceleration of tRNA selection. After decoding, EF-Tu dissociates from ribosome in the form of EF-Tu∙GDP, followed by the complete accommodation of aa-tRNA into the A-site. EF-Tu∙GDP is recycled to EF-Tu∙GTP by EF-Ts, a guanosine nucleotide-exchange factor, so as to participate in multiple rounds of peptide chain elongation. The next step is peptidyl transfer and peptide bond formation, which is catalyzed by the peptidyl transfer center (PTC) of the ribosome. During this stage, the nucleophilic α-amino group of the aa-tRNA in the A-site attacks the carbonyl carbon of the peptidyl-tRNA in the P-site, yielding a pre-translocation (PRE) ribosome complex with a deacylated tRNA in the P-site and a new, one residue longer peptidyl-tRNA in the A-site (Beringer and Rodnina, 2007). The third stage is EF-G∙GTP (eEF2∙GTP in eukaryotes) catalyzed translocation. Upon the addition of EF-G into the pre-translocational ribosome (PRE) system, EF-G in complexed with GTP facilitates movements of peptidyl-tRNA on the 50S subunit, and shifts the classical pre-translocation state to the hybrid state (Holtkamp et al., 2014a; Xie, 2016). And GTP hydrolysis induces a strong conformational change of 30S subunit within the 70S ribosome, allowing the movement of tRNA_2_-mRNA by one codon length inside the ribosome, shifting the tRNAs from A- and P-sites to P- and E-sites, respectively, resulting in a post-translocational ribosome (POST) (Figure 2).

There is a complex set of protein factors including EF-Tu/eEF1A, EF-G/eEF2, EF-P/eIF5A, and SelB/EFsec (Table 1), involved in translation elongation. Although the fundamental mechanism of the elongation cycle is very similar among three domains of life, the molecular mechanism of the elongation factors varies in different species. In the following, we will introduce in more detail the functions and regulation of the most important and well-studied canonical and non-canonical translation elongation factors that function in different stages of the elongation cycles of bacteria, archaea, and eukaryotes as well as that of organelles, including mitochondria.

**TABLE1:** Translation elongation factors among bacteria, archaea, eukaryotes and mitochondria.

Species	aa-tRNA incorporation			tRNA translocation		Other translation elongation factors				
Bacteria	EF-Tu	EF-Ts	SelB	EF-G	EF4	EF-P	—	Tet(O)/(M)	RelA	BipA
Archaea	aEF1A	—	—	aEF2	—	aIF5A	—	—	—	—
Eukaryotes	eEF1A	eEF1B	EFsec	eEF2	mtEF4	eIF5A	eEF3	—	—	—
Mitochondria	mtEF-Tu	mtEF-Ts	—	mtEF-G1	mtEF4	—	—	—	—	—

None.

## Factors Involved in AA-tRNA Incorporation

### EF-Tu and EF-Ts

EF-Tu is encoded by two genes *tufA* and *tufB* in *E. coli* (Jaskunas et al., 1975), *T. thermophilus* (Satoh et al., 1991) and *S. typhimurium* (Hughes, 1990). It is a universally conserved GTPase in all species (Caldon and March, 2003). During translation elongation, EF-Tu∙GTP transports aa-tRNA to the ribosome A-site in the form of the ternary complex (Ramakrishnan, 2002). Upon binding of the ternary complex to the ribosome, proper base pairing between the anticodon of aa-tRNA and the mRNA codon within the 30S subunit decoding region stimulates EF-Tu to hydrolyze GTP. After the hydrolysis of GTP, the conformational change following the GTP hydrolysis to GDP and a leaving phosphate group (Pi) leads to the dissociation of EF-Tu from the ribosome and accommodation of the aa-tRNA on the A-site for a peptidyl transfer (Rodnina et al., 2005). The growing peptide chain extends by one amino acid under the catalysis of the ribosome. Recycling of EF-Tu∙GDP to EF-Tu∙GTP depends on EF-Ts, another elongation factor encoded by the *tsf* gene (Wang et al., 1997). EF-Tu can be reversibly phosphorylated on its serine and threonine residues, and this modification has been founded in multiple organisms including *E. coli* (Lippmann et al., 1993), *Listeria monocytogenes* (Archambaud et al., 2005), *Thermus thermophilus* (Lippmann et al., 1993), *Streptococcus pneumoniae* (Sun et al., 2010), *Bacillus subtilis* (Lévine et al., 2006), *Corynebacterium glutamicum* (Bendt et al., 2003), *Mycoplasma pneumoniae* (Schmidl et al., 2010), *Mycobacterium tuberculosis* (Sajid et al., 2011), and *Streptomyces collinus* (Mikulík and Zhulanova, 1995). Phosphorylation inhibits the GTPase activity of EF-Tu and prevents its dissociation from the ribosome (Pereira et al., 2015). Phosphorylated EF-Tu could not bind with aa-tRNA or kirromycin (Hughes, 2013). It has been reported that phosphorylation of EF-Tu plays a vital role in bacterial dormancy, sporogenesis, virulence, and stress tolerance (Archambaud et al., 2005; Holub et al., 2007; Misra et al., 2011; Pereira et al., 2015). The exact physiological significance of EF-Tu phosphorylation is still need to be clarified while phosphorylation of EF1A in eukaryotes has been shown to be involved in maintaining a proper elongation rate under various conditions (Hughes, 2013).

### eEF1A and eEF1B

The GTPase eEF1A, the homolog of EF-Tu in bacteria, is one of the most widely expressed factors in eukaryotes (Schuller and Green, 2018). Human eEF1A has 33% sequence identity with bacterial EF-Tu (Cavallius et al., 1993). Similar to EF-Tu, another guanine nucleotide-exchange factor eEF1B is required to regenerate active eEF1A∙GTP (Gromadski et al., 2007). In lower eukaryotes, eEF1B contains a guanine nucleotide exchange subunit eEF1Bα and a structural subunit eEF1Bγ, while higher eukaryotes have another guanine nucleotide exchange subunit eEF1Bδ (plants) or eIF1Bβ (mammals). The mechanism of guanine-nucleotide exchange employed by eEF1B is very different from that of EF-Ts (Rodnina and Wintermeyer, 2009). Upon binding of EF-Ts to EF-Tu, the switch I region of EF-Tu is displaced by the C-terminal helix of EF-Ts. The switch II region is moved upon binding due to pushing by subdomain N. Altogether, these changes disrupt the coordination of the Mg^2+^ ion, leading to the dissociation of GDP. In contrast, eEF1Bα interacts with domains 1 and 2 of eEF1A, disrupting the binding pocket for Mg^2+^ and preventing the binding of the GDP to eEF1A.

**FIGURE 3:**
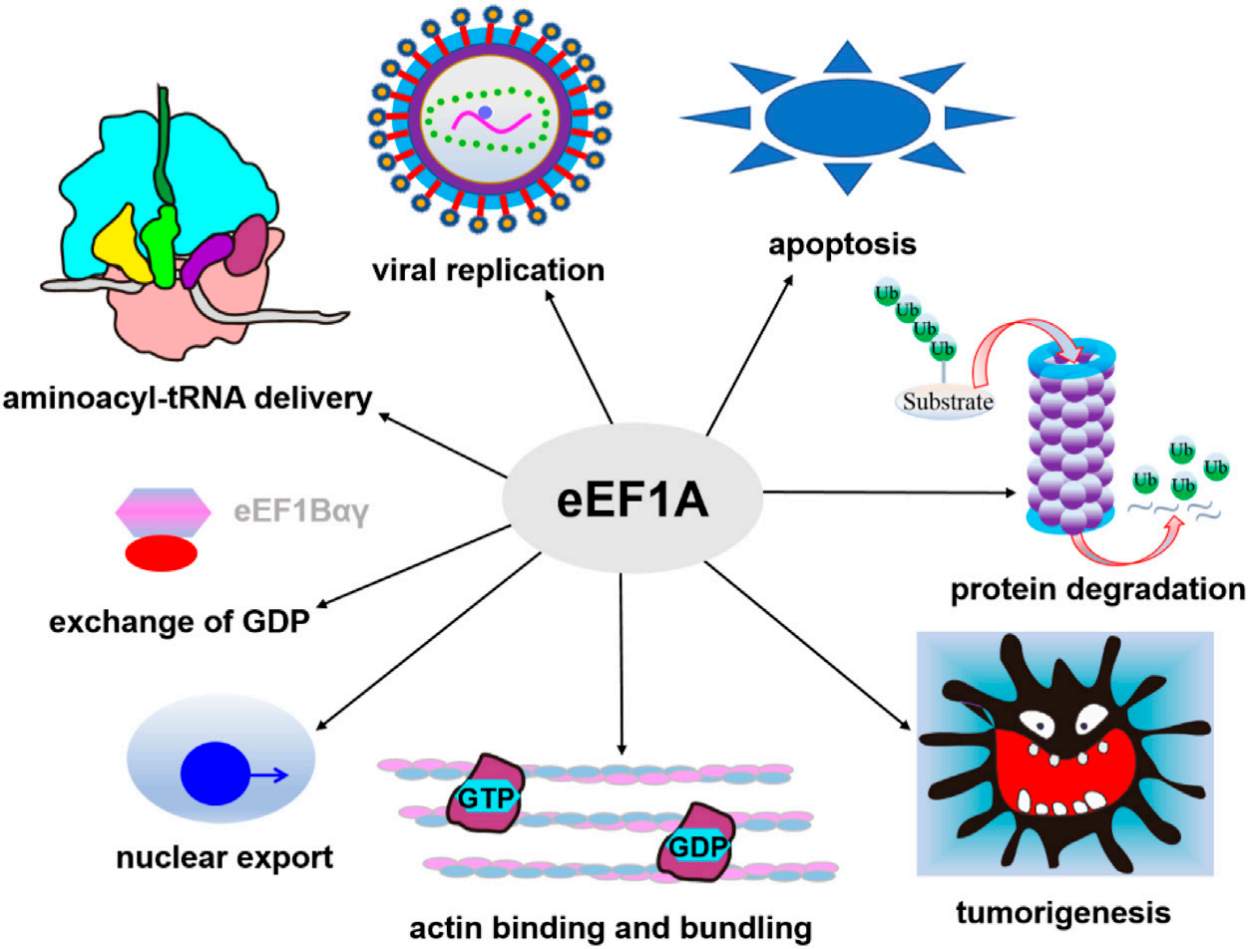
Canonical and non-canonical functions of eEF1A. In addition to its canonical functions in transporting aa-tRNA, eEF1A is also involved in cytoskeleton organization, protein degradation, viral replication and propagation, nuclear protein export, heat shock stress response, and signaling transduction pathway concerning apoptosis and oncogenesis.

In humans, there are two eEF1A homologues, named eEF1A1 and eEF1A2 (coded by two genes: *EEF1A1* and *EEF1A2*). The sequences of eEF1A1 and eEF1A2 have 98% similarity and 92% identity, but the expression patterns of the two proteins are different (Tomlinson et al., 2005). eEF1A1 is ubiquitously expressed, whereas the expression of eEF1A2 is switched-on in adult life in specialized tissues such as skeletal muscle, cardio-myocytes and neurons (Lee and Surh, 2009). Overexpression of eEF1A2 has been reported to be linked with a variety of tumors (Lee and Surh, 2009), and mutations in *EEF1A2* are related to a new type of epilepsy syndrome and intellectual disability (Inui et al., 2016; Lam et al., 2016). In addition to its canonical functions in transporting aa-tRNA to the ribosome, eEF1A is found to be involved in cellular activities such as regulation of cytoskeleton organization (Mateyak and Kinzy, 2010), protein degradation mediated by the proteasome (Mateyak and Kinzy, 2010), viral replication and propagation (Li et al., 2009), nuclear protein export (Khacho et al., 2008), signaling transduction pathway concerning apoptosis and oncogenesis (Schulz et al., 2014; Abbas et al., 2015) (Figure 3). eEF1A1 also plays an important role in the process of heat shock stress response (Vera et al., 2014). Single-cell transcriptomic analysis revealed that the expression level of eEF1A1 in neurons was low and changed with age in glial cells (Ximerakis et al., 2019). Therefore, eEF1A may represent a potential candidate for lifespan modulation (Skariah and Todd, 2020). In addition, eEF1A, along with eEF2, has been shown to be related to neurodegenerative disorders including Alzheimer disease (AD) and Parkinson disease (PD) with an unknown mechanism. The low expression levels of these factors in the brains of AD and PD patients indicating defects in the efficiency or fidelity of translation (Li et al., 2005; Vera et al., 2014; Garcia-Esparcia et al., 2015; Beckelman et al., 2016; Skariah and Todd, 2020). eEF1A has chaperone-like activity (Lukash et al., 2004) and may also be involved in antiviral response by interaction with Sgt1, a multifunctional protein (Novosylna et al., 2015).

Humans and yeast eEF1A are subjected to extensive methylation modifications at multiple conserved lysine residues (Hamey and Wilkins, 2018; Jakobsson et al., 2018; Robichaud et al., 2019). Methylation modification is the addition of 1∼3 (me1-me3) methyl groups to the side chains of lysine residues. Up to now, several methylation sites and corresponding methyltransferases of eEF1A have been identified in humans and yeasts. For example, human N6AMT2 (eEF1A-KMT1), METTL10 (eEF1A-KMT2), METTL21B (eEF1A-KMT3), and eEF1A-KMT4 (gene name *EEF1AKMT4*), which methylate eEF1A at K79 (me3), K318 (me3), K165 (me1/me2/me3), and K36 (me3), respectively (Shimazu et al., 2014; Hamey et al., 2016; Jakobsson et al., 2017; Malecki et al., 2017); In addition, two other methylation sites (N terminus and K55) have been reported in human cells, but the enzymes responsible for N-terminal trimethylation and K55 dimethylation in human eEF1A still need to be identified (Hamey et al., 2017). *Saccharomyces cerevisiae* Efm1, Efm4, Efm5, Efm6, and Efm7, which methylate eEF1A at K30 (me1), K316 (me2), K79 (me3), K390 (me1), and N-terminal (me3) and K3 (partially me1 and me2) respectively (Lipson et al., 2010; Dzialo et al., 2014; Jakobsson et al., 2015; Hamey et al., 2016). The abundant lysine methylations of eEF1A and the existence of multiple corresponding methyltransferases in various eukaryotes made it clear that the lysine methylation of eEF1A has important physiological significance. In *METTL21B* knockout (KO) human cells, the expression of proteins involved in cytoskeleton organization was downregulated, while the expression of proteins related to large ribosomal subunit biogenesis, mRNA turnover and rRNA processing were upregulated (Hamey et al., 2017). Moreover, mammalian METTL21B was found to be partially localized in the centrosome, which may reveal a non-canonical function for this protein (Malecki et al., 2017). In *EEF1AKMT4* KO cells, the global translation is changed and the translation speed of codons for histidine (H), tryptophan (W), and asparagine (N) was altered compared with wild-type cells (Jakobsson et al., 2017). In *EEF1AKMT1* KO cells, the expression of proteins related to tRNA aminoacylation and nuclear exosome were downregulated, while the expression of proteins related to ubiquitination regulation and small-subunit processome were upregulated (Hamey et al., 2017). Even though the physiological function of K318 methylation of eEF1A has not been clarified in mammalian, perhaps it can be speculated that K318 methylation may affect the replication of RNA virus and the migration of neural crest based on the highly conservative of this site between human and yeast (Shimazu et al., 2014). In yeast, Efm4 is involved in vesicle transport processes including secretory protein production, transfection and endocytosis (Martín-Granados et al., 2008). Efm4 is also play a vital role in Tombusvirus replication (Li et al., 2014). Similarly, the gene of *EFM5* was shown to be crucial for virus replication in yeast (Kushner et al., 2003). In *S. cerevisiae*, *EFM7* KO leads to decreased replicative lifespan (Anderson et al., 2003), which results from altered translation rate (Pan et al., 2007). Methylation of eEF1A by Efm6 occurs in its domain III, which is involved in protein translation and cytoskeleton organization (Gross and Kinzy, 2005; Liu et al., 2006). Finally, even though the five methyltransferases of eEF1A in *S. cerevisiae* are not absolutely necessary to its viability, the precise regulation of eEF1A function by distinct methyltransferases optimizes the cell physiology (White et al., 2019). Besides methylation, the lysine residues of eEF1A are also modified by acetylation, sumoylation and ubiquitination, as well as phosphorylation of tyrosine, threonine and serine residues (Hornbeck et al., 2012).

### mtEF-Tu and mtEF-Ts

Eukaryotic cells, including those in animals and fungi, contain two translation systems, one in the cytosol and the other in the mitochondria. Mitochondria use their own translational system to synthesize proteins for respiratory chain complexes. mtEFs (mitochondrial translation elongation factors) are coded by the nuclear genome, synthesized and transported into mitochondria. These factors are more similar to their counterparts in bacteria than those from the cytoplasm of eukaryotes. Genes encoding mtEFs such as *TUFM* (mtEF-Tu), *TFSM* (mtEF-Ts), and *GFM1*(mtEF-G1), have been reported with mutations in cases causing down regulation of mitochondrial translation and early fatality (Hughes, 2013).

mtEF-Tu consists of 409 amino acids, and is 55∼60% identical to the homologous protein from bacteria. It was found that mtEF-Tu folded into three main domains similar to EF-Tu. One of the main differences is that the C-terminal of mtEF-Tu has an 11 amino acids extension, which may interact with aa-tRNA (Jeppesen et al., 2005). Early studies showed that, mtEF-Tu was compatible with *E. coli* aa-tRNAs, whereas *E. coli* EF-Tu was unable to catalyze polypeptide chain elongation when supplied with mitochondrial aa-tRNAs (Kumazawa et al., 1991). This is probably due to the incorrect positioning of the shorter mitochondrial aa-tRNAs on the ribosome by bacterial EF-Tu, which leads to the ineffective stimulation of the GTPase activity of EF-Tu (Christian and Spremulli, 2012). Unlike EF-Tu, the bacterial and mitochondrial EF-Ts show low (25∼30%) sequence conservation. The most widely studied form of mtEF-Ts comes from *B. taurus*. It consists of 283 amino acids, with a mitochondrial import signal about 55 residues reside in the N-terminal of the mature protein (Xin et al., 1995).

### SelB

Selenocysteine (Sec) is a cysteine (Cys) residue analogue with a selenium-containing selenol group in place of the sulfur-containing thiol group in Cys. The selenium atom gives Sec quite different properties from Cys. Sec utilization is scattered across archaea (Mariotti et al., 2016) and bacteria (Zhang et al., 2006). In eukaryotes, selenoproteins exist in some algae and protozoa (Lobanov et al., 2009; Mariotti et al., 2015), and most metazoans (Mariotti et al., 2012). Recently, Mariotti et al. (2019) provided evidence for Sec usage in early-branching fungal phyla. In mammals, Sec exists in enzymes associated with ROS detoxification and hormone biosynthesis. It plays a vital role in many biological processes including development, reproduction, immune response, tumorigenesis, viral infections and cardiovascular diseases (Carlson et al., 2006). The terminator codon UGA behaves as the codon of Sec when the downstream of UGA possesses a selenocysteine insertion sequence (Ose et al., 2007). During the translation of selenoproteins such as glutathione peroxidase and bacterial formate dehydrogenase, SelB, a unique protein factor, is needed to deliver selenocysteinyl-tRNA^Sec^ containing a UCA anticodon to the ribosome A-site to recognize UGA codon in proper position of an mRNA (Böck et al., 1991). Incorporation of Sec is governed by a unique mRNA hairpin that located 3′ near the Sec codon (Yoshizawa et al., 2005). This hairpin structure associates with the corresponding ternary complex that is composed of SelB, Sec-specific aa-tRNA and GTP (Yoshizawa et al., 2005). It is assumed that by this mechanism selenocysteinyl-tRNA^Sec^ is delivered to the ribosome.

## Factors Involved in Ribosome Translocation

### EF-G

EF-G, which is the third most conserved trGTPase among all domains of life (Caldon and March, 2003), catalyzes the translocation of A-site peptidyl-tRNA and P-site deacylated tRNA to the P- and E-site, respectively (Ramakrishnan, 2002). Translocation of tRNA_2_-mRNA during translation elongation is associated with EF-G triggered GTP hydrolysis and a series of conformational changes of the ribosomes (Savelsbergh et al., 2005). Recently, Holtkamp et al. (2014b) concluded that EF-G integrates the energy regimes of a motor protein and a GTPase, and promotes tRNA motion through the combined use of power stroke and Brownian ratchet mechanisms. After GTP hydrolysis, EF-G departs from the ribosome in the form of EF-G∙GDP. EF-G is the only canonical trGTPase that functions at two distinct phases in bacterial translation (Ero et al., 2016). In addition to facilitating translocation in elongation, it also plays an essential role in ribosome recycling, during which EF-G∙GTP works together with RRF to split the post-termination complex (PoTC) into two subunits (Song et al., 2020).

The GTPase activity of EF-G involves two hydrophobic amino acids Ile61 and Ile19 (*E. coli* numbers), which facilitate approaching of His92 to GTP by forming an opened hydrophobic gate. The water molecule promoted by His92 attacks the γ phosphate of GTP, resulting in an active state of the GTPase center (Yamamoto et al., 2014). EF-G is extensively modified by reversible phosphorylation. An early study reported that *E. coli* EF-G can be phosphorylated by a serine/threonine specific protein kinase (gp 0.7 PK) encoded by the T7 early gene 0.7 (Robertson et al., 1994). This gene was expressed early following T7 infection, leading to rapid shutdown of host RNAP at 4 min after infection, and soon thereafter, most protein synthesis began to turn off gradually. Which established favorable conditions for T7 phage growth (McAllister and Barrett, 1977). And this modification may help to increase the translation elongation rate of T7 late genes that specify T7 virion assembly and structural proteins (Robertson et al., 1994). Another *in vivo* and *in vitro* study in *B. subtilis* showed that EF-G can be phosphorylated on at least one threonine residue by a membrane Ser/Thr kinase PrkC and dephosphorylated by phosphatase PrpC, and the dynamic control of EF-G phosphorylation may play a regulatory role in stationary-phase *B. subtilis* (Gaidenko et al., 2002). Later, Shah and Dworkin (2010) proved that phosphorylation of EF-G by PrkC in *B. subtilis* is in response to cell wall-derived muropeptides. *E. coli* EF-G can also be modified at the lysine residue essential for GTP binding by pyridoxal phosphate (PLP), a selective, site-specific lysine reagent, leading to progressive loss of the EF-G activity, and destruction of its interaction with 30S subunits as well as a conformational change required for GTP hydrolysis (Giovane et al., 1982). Although these effects have been more widely studied, the physiological significance of phosphorylated EF-G still needs to be elucidated (Hughes, 2013).

### eEF2

eEF2 is the eukaryotic homolog of EF-G. When cells are starved of nutrients, eEF2 is phosphorylated by the Ca^2+^-activated kinase eEF2K, resulting in a lower binding affinity to the ribosome (Carlberg et al., 1990). The activity of eEF2K is regulated by nutrients through mTORC1 and AMPK (Kenney et al., 2014; Proud, 2019). During translation elongation, the active eEF2K can reduce termination read-through errors and codon-anticodon mismatches, and promote more accurate recognition of the start codon by reducing initiation at the near-AUG codons (Xie et al., 2019). Interestingly, despite the major role of eEF2’s phosphorylation in blocking whole protein translation, its phosphorylation in neurons is associated with elevated translation of Arc/Arg3.1 which plays a key role in postsynaptic endocytosis (Park et al., 2008).

**FIGURE 4:**
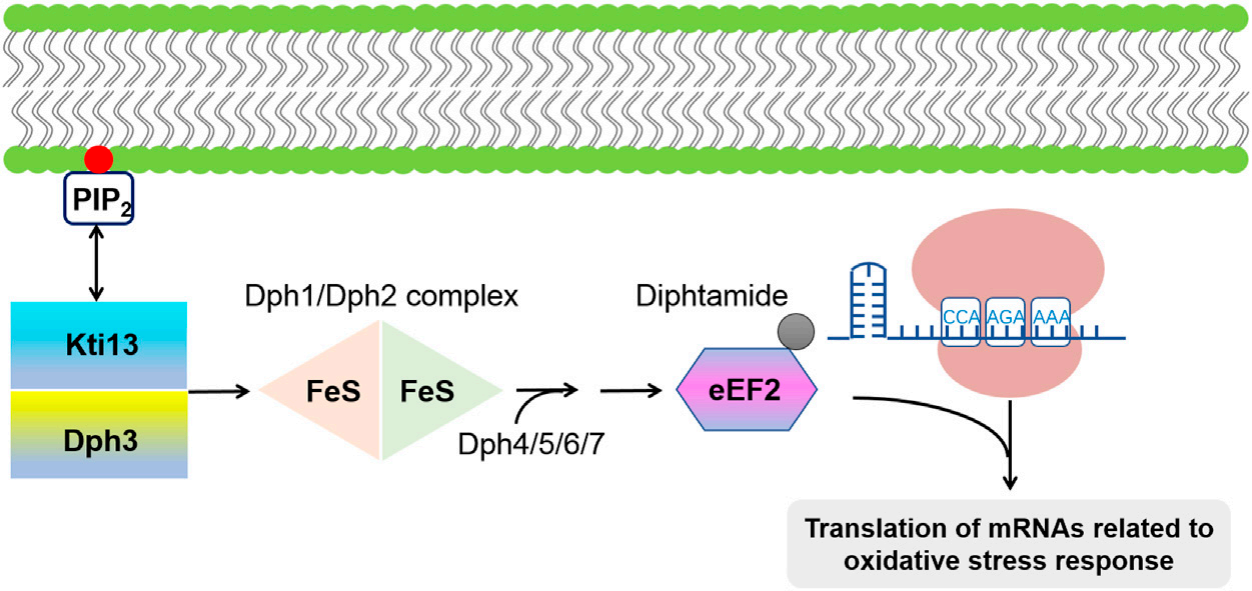
Schematic of the diphtamide modification of eEF2. The first step in this modification pathway is catalyzed by [4Fe-4S] cluster-containing protein complex Dph1/Dph2; with the help of Dph4/5/6/7. Dph3/Kti13 heterodimer act as electron donor for Dph1/Dph2 complex. It has been reported that diphtamide group of EF2 can further be ADP-ribosylated by the bacterial diphtheria toxin, leading to a global inhibition of protein synthesis as well as an upregulated translation of mRNAs associated with oxidative stress response.

Diphthamide is another conserved modification in archaeal and eukaryal eEF2, where a conserved histidine (H715 in mammals; H699 in *S. cerevisiae*) at the eEF2 domain IV is modified with diphthamide (Su et al., 2013; Schaffrath et al., 2014). In eukaryotes, this modification event occurs through a four-step pathway involving multiple proteins including Dph1-Dph7 (Versées, 2015). Dph1/Dph2 is a [4Fe-4S] cluster-containing heterodimeric protein complex, which is responsible for catalyzing the first step of this modification pathway. In *S. cerevisiae*, the highly conserved proteins Dph3 (or Kti11) and Kti13 form a heterodimer, which is involved in eEF2 modification by acting as an electron donor for the Dph1/Dph2 complex (Dong et al., 2014; Glatt et al., 2015). With the help of Dph4/5/6/7, the diphthamide group was finally added to eEF2. Moreover, Kti13 was reported to specifically binding with PIP_2_ (Di Paolo and De Camilli, 2006), which might contribute to the regulation of the downstream eEF2 modification pathway (Figure 4). Lack of the diphthamide modification is fatal to mice due to severe developmental defects (Yu et al., 2014). It is worth noting that yeast lacking Dph1, an enzyme needed for diphthamide synthesis, grew normally, indicating that diphthamide likely functions in translation fidelity but not a basic mechanism of translation (Dever et al., 2018). Besides, some mammalian cells can survive in the absence of diphthamide (Stahl et al., 2015). However, related studies (Liu et al., 2012) had shown that lacking diphthamide modification results in an increase in the level of programmed -1 ribosomal frameshifting. Considering its location in eEF2, it is reasonable to speculate that diphthamide may enhance the eEF2 function by contacting with RNA in the ribosomal decoding center, thus facilitating the ribosomal translocation fidelity (Dever and Green, 2012).

Diphthamide of eEF2 can be further ADP-ribosylated by diphtheria and cholera toxins, which catalyze the transfer of ADP-ribose from nicotinamide adenine dinucleotide (NAD^+^) to the diphthamide imidazole ring to yield ADP ribosyl diphthamide (Argüelles et al., 2014). ADP-ribosylation inactivates eEF2, hinders protein translation and damages cell growth (Mateyak and Kinzy, 2013). Recently, it has been reported that cells possess intrinsic abilities to modify the diphthamide group by ADP-ribosylation, and this ability will improve under certain stress conditions, causing the overall down-regulation of protein synthesis at the cost of an increased translation of IRES-containing mRNA that involved in the response of oxidative stresses (Argüelles et al., 2014). However, the molecular mechanism of how ADP-ribosylation impairs the function of eEF2 has not been fully elucidated (Mateyak and Kinzy, 2013).

### mtEF-G1 and mtEF-G2

In human mitochondria, the dual function of bacterial EF-G is fulfilled by mtEF-G1 and mtEF-G2 (Tsuboi et al., 2009; Christian and Spremulli, 2012). During mitochondrial translation elongation, ribosome translocation was catalyzed by mtEF-G1. Previous studies revealed that mtEF-G1 has a strong tolerance to fusidic acid, an antibiotic that inhibits EF-G release from the ribosome without influencing on GTP hydrolysis and translocation (Gao et al., 2009; Savelsbergh et al., 2009), while the tolerance mechanism of mtEF-G1 to fusidic acid remains to be understood (Christian and Spremulli, 2012). mtEF-G1 is active not only with 55S mammalian mitochondrial ribosome but also with 70S bacterial ribosome. In contrast, *E. coli* EF-G can’t work with mitochondrial ribosomes (Chung and Spremulli, 1990). mtEF-G2 mediates ribosome recycling in concert with human mitochondrial RRF after termination (Achenbach and Nierhaus, 2015). However, it should be noted that overexpression of mtEF-G2 can improve the translation of respiratory chain complexes slightly in cells with mtEF-G1 mutation, indicating that mtEF-G2 perhaps plays a part in the translation elongation (Coenen et al., 2004). Unlike ribosome recycling in bacteria, mtEF-G2 catalyzed GTP hydrolysis is not essential for ribosome dissociation. Rather, it seems to be necessary for the dissociation of mtRRF and mtEF-G2 from ribosomes. Since mtEF-G2 represents a type of trGTPase participating in ribosome recycling, it has been proposed to rename this factor as mitochondrial ribosome recycling factor 2 (mtRRF2) (Tsuboi et al., 2009).

## Other Elongation Factors

### EF4

EF4 (LepA) was originally identified in *E. coli* in 1985 (March and Inouye, 1985). The high conservation of EF4 in bacteria suggests its functional importance (Margus et al., 2007). Nierhaus and co-workers reported that cell membranes behave as EF4 reservoir pool, releasing it to the cytoplasm under certain conditions such as elevated intracellular Mg^2+^ concentrations or low temperature, leading to an increased rate of translation and efficient folding of newly synthesized peptides (Pech et al., 2011; Yamamoto et al., 2014). Functional studies showed that EF4 knockout affects bacterial growth under conditions of high Mg^2+^ concentration (Pech et al., 2011) or low pH (Yang et al., 2014). In *S. cerevisiae*, lacking EF4 (Guf1) results in growth defects under conditions of starvation and low temperature, and decreased expression of cytochrome oxidase (Bauerschmitt et al., 2008). Besides, EF4 knockout *E. coli* cells showed a decreased translation rate and slow ribosome maturation at unfavorable conditions (Yang et al., 2014). Overexpression of EF4 in *E. coli* seriously affects the growth of cells (Qin et al., 2006), while EF4 knockout cells showed no obvious phenotype under culture conditions of rich LB medium (Shoji et al., 2010). Therefore, EF4 may contribute to the cell survival under adverse conditions, but the physiological role of the protein remains unclear.

A previous study proposed that EF4 acts as a ‘back-translocase’ that has a unique property of recognizing ribosomes with mistranslocated tRNAs and back-translocating them via GTP hydrolysis during elongation cycle (Qin et al., 2006). However, several subsequent studies could not confirm that EF4 did have this biochemical activity (Liu et al., 2010; Balakrishnan et al., 2014; Gibbs and Fredrick, 2018). Cooperman and coworkers have done the most detailed biochemical characterization of EF4, studying its effect on both PRE and POST state complexes (Liu et al., 2010; Liu et al., 2011). They found that the addition of EF4 to POST can promote movement of tRNA with respect to the 50S subunit but does not catalyze back-translocation. Notably, Cooperman also showed that the EF4 preferentially engages the PRE complex, and competes with EF-G rather than EF-Tu ternary complex to influence elongation. *In vitro* kinetic measurements showed that EF4-dependent back translocation proceeds through a four-stage kinetic route (POST→I_1_→I_2_→I_3_→PRE), not just a reversal of translocation but exist three intermediate states, and the rate of reverse codon-anticodon movement observed in the presence of EF4 is virtually identical to that seen in its absence (Liu et al., 2010), in line with the independent work led by Fredrick and coworkers, whose ribosome profiling results revealed that EF4 contributes mainly to the initiation phase of translation in *E. coli* (Balakrishnan et al., 2014). This is consistent with a recent physiological study suggesting EF4 contributes to biogenesis of the 30S subunit, immature 30S particles will accumulate in cells lacking EF4 (Gibbs et al., 2017).

**FIGURE 5:**
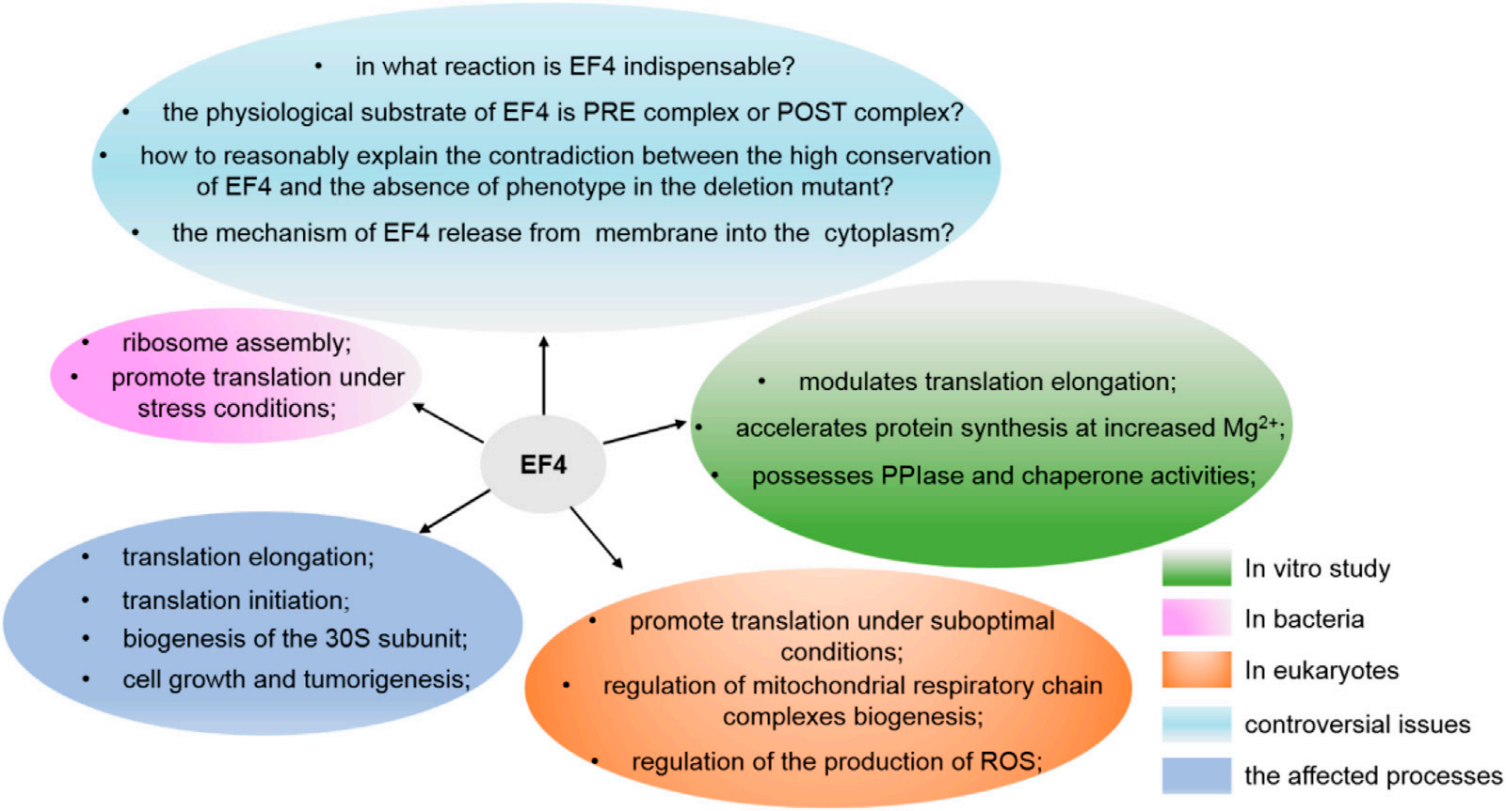
Various functions of EF4 and the issues still need to be resolved.

In summary, although the major role of EF4 is to facilitate bacteria in response to some stresses and affect protein synthesis in general, still some important issues concerning EF4 need to be resolved: What is the mechanism of EF4 release from the membrane into the cytoplasm? What is the real physiological substrate of EF4? How to reasonably explain the contradiction between the high conservation of EF4 and the obvious lack of phenotype in its deletion mutants (Figure 5)?

### mtEF4

mtEF4 is the homologue of bacterial EF4 in eukaryotic mitochondria. It consists of 651 residues with a mitochondrial-targeting signal in their N termini (Gao et al., 2016). mtEF4 is located in the mitochondrial matrix, close to the inner membrane (Bauerschmitt et al., 2008). mtEF4 binds to the ribosome in a GTP-dependent manner which is similar to that of bacterial EF4. It promotes the translation of mitochondrial proteins under non optimal conditions (Bauerschmitt et al., 2008). mtEF4 also plays a quality control role in the biogenesis of mitochondrial respiratory chain complexes. Previous studies showed that mtEF4 knockout induces respiratory chain defects as well as apoptosis, whereas overexpression of the protein stimulates cancer development (Zhu et al., 2018). mtEF4 ablation in mice results in testis-specific disorder of oxidative phosphorylation, and its deletion facilitated mitochondrial protein translation in the expense of synthesis unstable proteins (Gao et al., 2016). Increased expression of mtEF4 in multiple cancers suggested that mtEF4 probably facilitates tumorigenesis through an unbalanced regulation of mitochondrial activities and cellular redox (Zhu et al., 2018). Therefore, the proper level of mtEF4 in cell is requisite for the assembly of functional respiratory chain complexes as well as mitochondrial protein synthesis.

### EF-P and eIF5A

Bacterial EF-P (elongation factor P) is a homolog of eukaryotic and archaeal initiation factor 5A(e/aIF5A) (Kyrpides and Woese, 1998). It binds to the interface of the ribosome subunits and facilitates peptide bond formation through interactions with the P-site tRNA (Glick and Ganoza, 1975; Blaha et al., 2009). *efp*, the coding gene of EF-P, has been found throughout the bacteria (Hughes, 2013). It was reported that EF-P participates in the regulation of cell viability, growth, motility, virulence (Ude, 2013), and tolerance to multiple stresses including several classes of antibiotics, detergents, nutrient-limiting conditions and diverse growth inhibitors (Navarre et al., 2010; Zou et al., 2012). The ribosome needs the help of EF-P when successive prolines are incorporated into the nascent peptide chain (Rodnina, 2018). Without EF-P, the ribosome would stagnate at polyproline stretches, while the addition of EF-P could alleviate the translation stalling (Doerfel et al., 2013). EF-P is often subject to post-translational modification by PoxA catalyzed adding of a (*R*)-β-lysine to Lys34 (termed lysinylation) in bacteria like *E. coli* (Peil et al., 2012), *S. enterica* (Hersch et al., 2013) and *S. typhimurium* (Navarre et al., 2010; Zou et al., 2011), which could improve the catalytic activity of EF-P both *in vitro* and *in vivo* (Navarre et al., 2010; Park et al., 2012). β-lysylated EF-P also undergoes further hydroxylation, a second post-translational modification of this factor, but the function of hydroxylation does not seem to be critical for EF-P (Peil et al., 2012; Bullwinkle et al., 2013). In addition to the post-translational modifications mentioned above, other modifications of EF-P had been identified in other bacteria. For example, rhamnosylation of Arg32 in *S. oneidensis*, *P. aeruginosa*, *N. gonorrhoeae*, *B. pertussis* and *N. meningitidis*, which is closely related to bacterial fitness, pathogenicity and viability (Lassak et al., 2015; Rajkovic et al., 2015; Yanagisawa et al., 2016; Hummels and Kearns, 2020); 5-aminopentanolylation of Lys32 in *B. subtilis*, which can regulate the synthesis of diprolyl motifs of specific proteins needed for swarming motility (Rajkovic et al., 2016). There are hundreds of polyproline-containing peptides and proteins with different functions in all organisms, indicating EF-P (e/aIF5A) is necessary for the regulation of expression levels in various pathways.

**FIGURE 6:**
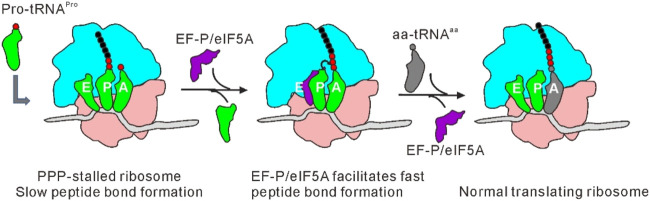
Mechanism of EF-P/eIF5A in alleviating ribosome stalling at polyproline stretches. Accomodation of the third consecutive Pro-tRNA^Pro^ into the A-site of translating ribosome leads to stalling. EF-P/eIF5A binds to a location between E- and P-sites of the stalled ribosome after E-site tRNA is dissociated, stimulating rapid proline-proline peptide bond formation. Translation can resume following dissociation of EF-P/eIF5A and binding of the next aa-tRNA.

eIF5A was firstly reported to promote the first peptide bond formation and was denoted as an initiation factor (Kemper et al., 1976). In archaea and eukaryotes, a conserved lysine located at the eIF5A domain I (Kim et al., 1998) is modified to hypusine post-translationally. Synthesis of hypusine is catalyzed by two consecutive enzymatic reactions involving deoxyhypusine synthase (DHPS) and deoxyhypusine hydroxylase (DOHH) (Park et al., 2010). Hydroxyl radical probing experiments revealed that the binding site of eIF5A on the ribosome is adjacent to the E-site, and the hypusine group is in proximity to the acceptor arm of the P-site tRNA (Gutierrez, 2013). An early study showed that unmodified eIF5A is unable to catalyze the formation of methionyl-puromycin (Park et al., 1991). Depletion of eIF5A leads to dysregulated translation initiation (Manjunath et al., 2019), elongation/termination (Schuller et al., 2017), and an increase in ribosomal transit times (Henderson and Hershey, 2011). In the absence of cycloheximide, inhibition of an eIF5A mutant that is sensitive to temperature leads to polysome accumulation (Saini et al., 2009), indicating eIF5A plays a vital role in the elongation phase. Moreover, if eIF5A is present, ribosome stalling will be restored when multiple prolines are to be incorporated *in vitro*. eIF5A depletion leads to defects in the synthesis of polyproline-containing proteins (Gutierrez, 2013), and ribosome stalling at tripeptides such as RDK, DVG, DDG, DDP, PDP and DNP (Schuller et al., 2017). Therefore, like EF-P in bacteria, eIF5A is considered to promote peptide transfer and improve the translation efficiency of poor substrates like proline (Gutierrez, 2013) (Figure 6). eIF5A was also found to play a role in the start codon selection during translation initiation. Depletion of eIF5A enhances upstream translation within 5′ UTRs across yeast and human transcriptomes (Manjunath et al., 2019).

### eEF3-Fungal Specific Elongation Factor

eEF3, a third elongation factor which was reported to be important for the protein translation and viability in higher fungi including yeast and *P. carinii* (Triana-Alonso et al., 1995). eEF3 from *S. cerevisiae* is composed of 1,044 amino acids and the coding gene of this factor is *YEF-3* (Qin et al., 1990). The crystal structure of *S. cerevisiae* eEF3 revealed that it is composed of a HEAT domain, two ATPase domains, a four-helix bundle, and a chromodomain (Andersen et al., 2006). It has a ribosome-dependent ATPase and GTPase (Dasmahapatra and Chakraburtty, 1981). eEF3 is mainly associated with cytosolic polysomes, and is needed for peptide bond formation (Kapp and Lorsch, 2004). The existence of eEF3 exclusively in fungi may be the most striking exception to the highly conserved translation elongation. It was reported that eEF2 and eEF1 from yeast can work with mammalian ribosomes to promote translation. In contrast, eEF2 and eEF1 from mammalian could function with yeast ribosomes only when eEF3 is present (Skogerson and Engelhardt, 1977), indicating eEF3 is required for yeast ribosome translation. Cryo-EM structures revealed that eEF3 has a new binding site close to the ribosome E-site, with the chromodomain stabilizing L1 stalk to facilitate tRNA release, which is consistent with the model that eEF3 may aid E-site tRNA release following translocation (Andersen et al., 2006).

The question that still needs to be answered is why do fungal ribosomes require eEF3 to promote E-site tRNA release and A-site occupancy while other ribosomes do not? Rodnina et al. (1994) reported that 80S from several higher eukaryotes can hydrolyse ATP and GTP without protein factors. This inherent ATPase activity functions as eEF3, promoting the dissociation of deacylated tRNA at the ribosome E-site (El’skaya et al., 1997), and cognate tRNA binding at the ribosome A-site seems to stimulate this activity in turn (Rodnina et al., 1994). Nevertheless, no homologues of fungal eEF3 have been found in mammalian cells. It is worth noting that a crystal structure of *S. cerevisiae* 80S contains an additional non-ribosomal protein, Stm1, which bound to the 40S subunit and precluded mRNA entry by placing a α-helix to the mRNA tunnel (Ben-Shem et al., 2011). In yeast, deletion of *stm1* leads to increased binding affinity of eEF3 to ribosomes, whereas up-regulation of eEF3 expression in cells lacking Stm1 leads to growth defect and elevated anisomycin (a translation inhibitor) sensitivity. Moreover, a high level of Stm1 in ribosomes displays reduced eEF3 binding. Therefore, it can be concluded that Stm1 and eEF3 may jointly promote the elongation cycle (Van Dyke et al., 2009). Even though the characteristics of bacterial ribosome structures do not suggest the need for another elongation factor (Dever and Green, 2012), *E. coli* has an ATPase RbbA, which can bind to the ribosome and stimulates protein translation *in vitro*, and displays multiple biochemical properties similar to that of eEF3 (Kiel and Ganoza, 2001). More studies concerning biochemistry, structural biology and high resolution fluorescence imaging are required to fully reveal the function of eEF3 and to resolve its specific needs in fungi translation elongation (Dever and Green, 2012).

### Tet(O) and Tet(M)

The tetracycline resistance proteins (Tet), which protect the bacterial ribosome from binding the antibiotic tetracycline, are another class of ribosome-associated GTPases (Margus et al., 2007). They are also called ribosomal protection proteins (RPPs). They are cytoplasmic proteins that display homology with the elongation factors EF-Tu and EF-G (Hughes, 2013). They bind to the ribosome, hydrolyze GTP and cause the release of tetracycline from the ribosome (Connell et al., 2003). RPPs are paralogs of elongation factors, and the best characterized and widely distributed RPPs are Tet(M) and Tet(O) (Hughes, 2013). Both Tet(M) and Tet(O) have ribosome-dependent GTPase activity, the hydrolysis of GTP providing the energy for the ribosomal conformational changes (Connell et al., 2003). Direct competition experiments showed that the ribosome binding site of Tet(M) is overlapping with that of EF-G (Dantley et al., 1998). To clarify the mechanism of the tetracycline resistance, Burdett purified Tet(M) protein (Burdett, 1991) and investigated its effects on several reactions that occur during protein translation (Burdett, 1996). The author found that Tet(M) could alleviate the inhibition of tetracycline on factor-dependent tRNA binding, and significantly reduce the affinity of ribosomes for tetracycline in the presence of GTP. Adding Tet(M) to the ribosome-tetracycline complex will replace the bound tetracycline. Therefore, the dissociation of tetracycline from the ribosome promoted by Tet(M) is GTP dependent. Structures of 70S∙Tet(O) complex revealed that Tet(O) really looks like EF-G and binds to the ribosome with an identical site (Spahn et al., 2001). In 2013, Li et al. identified the Tet(O) binding site on the ribosome, which involves three unique loops in Tet(O) domain IV (Li et al., 2013). The single glycine substitution of the residues in these loops leads to loss of tetracycline resistance.

### RelA

In order to adapt to the changes in environment conditions, pathogens have evolved a variety of mechanisms to respond to various stresses, the most important of which is called stringent response (SR) (Winther et al., 2018). SR is a nearly universal mechanism mediated by pppGpp and ppGpp (Steinchen and Bange, 2016). Seeing that SR is related to metabolic regulation, biofilm formation, virulence gene expression (Yang et al., 2020), stress, antibiotic resistance, as well as the formation ability of bacteria retention (Hauryliuk et al., 2015), therefore, the signal pathway becomes a target for designing effective antibacterial agents (Kushwaha et al., 2019). The intracellular levels of pppGpp and ppGpp (collectively referred to as (p)ppGpp) are controlled by RelA/SpoT Homologue (RSH) proteins, which synthetize (p)ppGpp by transferring the pyrophosphate group of ATPs onto the 3′ of GDP or GTP, and degrade (p)ppGpp by removing the 3′ pyrophosphate moiety. It interacts with the ribosome to sense environmental pressure and leads to an adaptive response of pathogens. When pathogens encounter stresses like nutrition deficiency, deacylated tRNA binds to the empty ribosome A-site to form the so-called “starving” ribosome, triggering the (p)ppGpp synthetic activity of RelA (Haseltine and Block, 1973). At this time, RelA catalyzes the production of AMP and ppGpp or pppGpp from ATP and GDP or GTP, respectively (Wendrich et al., 2002), and the SR reaction is triggered.

Under stressed conditions, ppGpp, as a “global transcriptional regulator”, can up regulate the expression of many genes at the transcriptional level, and the concentration of intracellular ppGpp is largely determined by *relA* gene. Therefore, the function of *relA* gene in cell physiology is mainly reflected in controlling the concentration of intracellular ppGpp. Studies have shown that (P)ppGpp can change the physiology of bacteria from rapid growth to slow growth, so as to allow them to survive under harsh conditions (Hauryliuk et al., 2015). In *E. coli*, (P)ppGpp combines with RNA polymerase, up-regulating the expression of metabolic enzyme genes, especially amino acid biosynthesis genes, while reducing the transcription of tRNA, rRNA, ribosomal protein, translation factor and synthase genes (Barker et al., 2001; Lemke et al., 2011; Ross et al., 2016). Other main effects include activating the stress factor δ^E^ and inhibiting cell wall synthesis (Costanzo et al., 2008). It was found that more than 30% of the genes in *E. coli* were differentially expressed by (P)ppGpp, including the up regulation of SR related genes and the down regulation of macromolecular structure related genes under isoleucine starvation conditions (Traxler et al., 2008). Several models have been proposed for the molecular mechanism of SR (Sanchez-Vazquez et al., 2019). It is reported that (P)ppGpp directly binds to RNAP with RNAP binding protein dksA, which destroys the stability of its open complex. On the other hand, (P)ppGpp indirectly regulates gene expression by δ competition (Sanchez-Vazquez et al., 2019). It is worth noting that almost all bacterial pathogens need SR, otherwise they cannot survive and infect the host during stress conditions.

### BPI-Inducible Protein A

BPI-inducible protein A (BipA) is a highly conserved ribosome-dependent trGTPase that regulates multiple cellular processes including bacterial attachment and effacement, motility, virulence gene expression, avoidance of host defenses. In addition, BipA is also associated with temperature, symbiosis, antimicrobial resistance and biofilm formation (Overhage et al., 2007; deLivron and Robinson, 2008; Neidig et al., 2013). BipA is widespread in most of the bacteria and plants (Margus et al., 2007) and is similar to EF4 and EF-G except for a unique C-terminal domain (Neidig et al., 2013; Kumar et al., 2015). Despite its conservation in bacteria, BipA was regarded as an essential factor only in adverse growth conditions such as low pH and temperature, nutrient deprivation, and other stresses (Starosta et al., 2014). Mutations in BipA leads to multiple phenotypes including cold sensitivity (Pfennig and Flower, 2001), hypermotility (Farris et al., 1998), decreased capsule synthesis (Rowe et al., 2000), increased sensitivity to chloramphenicol (Duo et al., 2008), and reduced pathogenicity (Grant et al., 2003). Moreover, it also participated in regulating some mRNAs translation under stresses (Ero et al., 2016). Interestingly, deLivron reported that BipA has two different binding modes to the ribosome. It is associated with 70S in the form of GTP-bound under normal cellular conditions, whereas it interacts with 30S subunit under stress conditions (deLivron and Robinson, 2008). Therefore, they speculated that there exist two ribosome∙BipA complexes that affect the response of bacteria to environmental conditions (deLivron and Robinson, 2008). Moreover, there is a growing body of evidence that BipA also functions in the 50S ribosomal subunit biogenesis (Choudhury and Flower, 2015; Del Peso Santos et al., 2021). Choi et al. (2019) reported that BipA is vital for 50S biogenesis at a low temperature, whose expression is involved in the incorporation of L6 protein. The exogenous expression of the L20 coding gene *rplT* can partially repair the defects in rRNA processing and ribosomal assembly, and then restore the growth of *bipA*-deficient strains at low temperatures. So, the authors speculated that BipA and L20 may play a coordinating role in proper ribosomal assembly under cold-shock conditions. Another study led by Fredrick (Gibbs et al., 2020) demonstrated the flexible nature of the 50S assembly process. They also found GTP hydrolysis was crucial to the function of BipA.

## Elongation Factors and Tumorigenesis

Recently, more and more elongation factors have been reported to function as oncoproteins or tumor promoters in cancer cells (Table 2). Elevated levels of protein synthesis are a critical feature of tumor cells. Alterations in protein synthesis can increase the overall translation rate and stimulate the translation of certain mRNAs to facilitate tumorigenesis, oncological progress, and survival.

**TABLE 2:** Alterations of translation elongation factors in human cancer.

Elongation factors	Observed modification	Association with cancer
eIF5A1	Increased expression	eIF5A is highly overexpressed in patients with glioblastoma (Preukschas et al., 2012). eIF5A1 is a diagnostic marker of vulvar intraepithelial neoplasia (Cracchiolo et al., 2004). eIF5A1 is upregulated in colorectal adenoma (Lam et al., 2010)
eIF5A2	Increased expression	eIF5A2 is amplified in ovarian carcinoma (Guan et al., 2001) and is associated with metastatic progression in colorectal cancers (Xie et al., 2008)
		Gain of *EIF5A2* results in recurrence of hepatocellular carcinoma (Chen et al., 2000)
		Ectopic expression of *EIF5A2* causes tumorigenesis in naked mice (Guan et al., 2004)
		Overexpression of eIF5A2 results in local invasion of non-small-cell lung cancer (He et al., 2011)
eEF1A1	Increased expression	eEF1A1 was upregulated in the infiltrating edge of invasive breast cancers (Zhu et al., 2003)
		Dimethylation of eEF1A is upregulated and can serve as a diagnostic marker for poor outcomes in lung and pancreatic cancer (Liu et al., 2019)
eEF1A2	Increased expression	eEF1A2 is distributed in 30% of ovarian cancers, and 26% of cancers with amplifications near the *EEF1A2*
		eEF1A2 acts as an oncoprotein which is upregulated in 67% of breast cancers (Tomlinson et al., 2005)
eEF2	Increased expression	eEF2 is overexpressed in most of colorectal and gastric tumors and promotes cancer growth *in vivo* and *in vitro* (Nakamura et al., 2009)

### eEF1A and Tumorigenesis

eEF1A is overexpressed in malignancies such as ovarian tumors. It can be inactivated by cytotoxic and anti-adipogenic ternatin and its derivatives (Carelli et al., 2015), with unclear mechanisms. Mutation in domain III of eEF1A hinders the binding of ternatin and confers resistance to its cytotoxic effects (Carelli et al., 2015). Cancers driven by the activation of PI3K-AKT axis are sensitive to the inhibitors of eEF1A (Lee and Surh, 2009). eEF1A1 was reported to be increased in the periphery of mammary cancer compared with the central region (Zhu et al., 2003), and the levels of eEF1A1 in neoplastic are relatively higher than in normal tissue (Lee and Surh, 2009). eEF1A2, an isoform of eEF1A, functions as cancer protein *via* a variety of mechanisms, such as facilitating cell invasion and migration by up-regulating MMP-9 and stimulating AKT in pancreatic tumors (Xu et al., 2013). It can accelerate proliferation and block apoptosis *via* down-regulating caspase 3 in prostate cancer tissues (Sun et al., 2014). Previous studies showed that the expression levels of eEF1A2 are elevated (Anand et al., 2002; Lee and Surh, 2009) and their genes (genomic region: 20q13) are amplified in a high proportion of solid tumors, such as ovarian (Iwabuchi et al., 1995) and breast cancers (Kallioniemi et al., 1994). For malignancy in the blood system, eEF1A2 is also up-regulated in cells of multiple myeloma (Losada et al., 2016). Therefore, eEF1A2 can be used as a diagnostic marker and target for some breast tumours (Tomlinson et al., 2005) and hematological malignancies (Shan et al., 2020).

**FIGURE 7:**
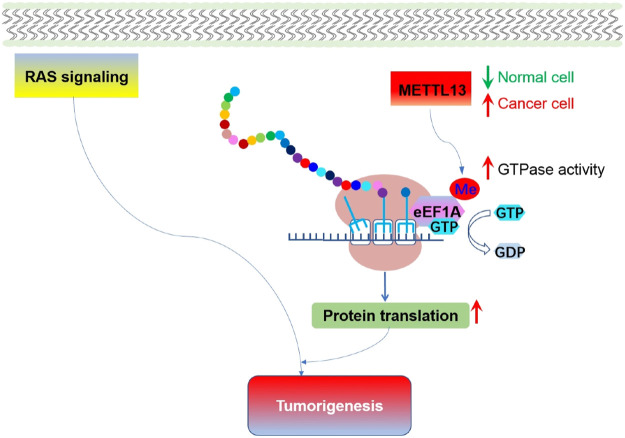
Methylation of eEF1A and the role of eEF1AK55me2 mediated translational control in tumorigenesis. Expression of METTL13 and eEF1AK55me2 are upregulated in cancer, and negatively correlate with the survival of pancreatic and lung cancer patient. Increased METTL13 and eEF1AK55me2 promotes Ras-driven tumorigenesis *in vivo*.

Besides phosphorylation, other post-translational modifications on elongation factors have been found to be essential for tumorigenesis. Recently, it was reported that lysine 55 of eEF1A is dimethylated (eEF1AK55me2) by METTL13 (Methyltransferase-like 13), resulting in an increase of the inherent GTPase activity of eEF1A (Liu et al., 2019). This modification of eEF1A was utilized by RAS signal cascade to promote translational output and facilitate carcinogenesis (Figure 7). Therefore, METTL13-eEF1AK55me2 signal pathway is vital for tumors to cope with increased translational demand, and METTL13 inhibition might be an effective way to do targeted intervention for RAS driven cancers.

### eEF2 and Tumorigenesis

eEF2 plays an essential role in many biological processes including cell cycle (Hizli et al., 2013) and genotoxic stress (Kruiswijk et al., 2012). Previous studies have shown that eEF2 is overexpressed in various tumors. Translation of eEF2 was up-regulated in most of colorectal (91.7%) and gastric (92.9%) tumors, leading to the elevation of *in vivo* tumorigenicity (Nakamura et al., 2009). Therefore, eEF2 is a potential target for tumor immunotherapy in multiple cancers (Oji et al., 2014).

**FIGURE 8:**
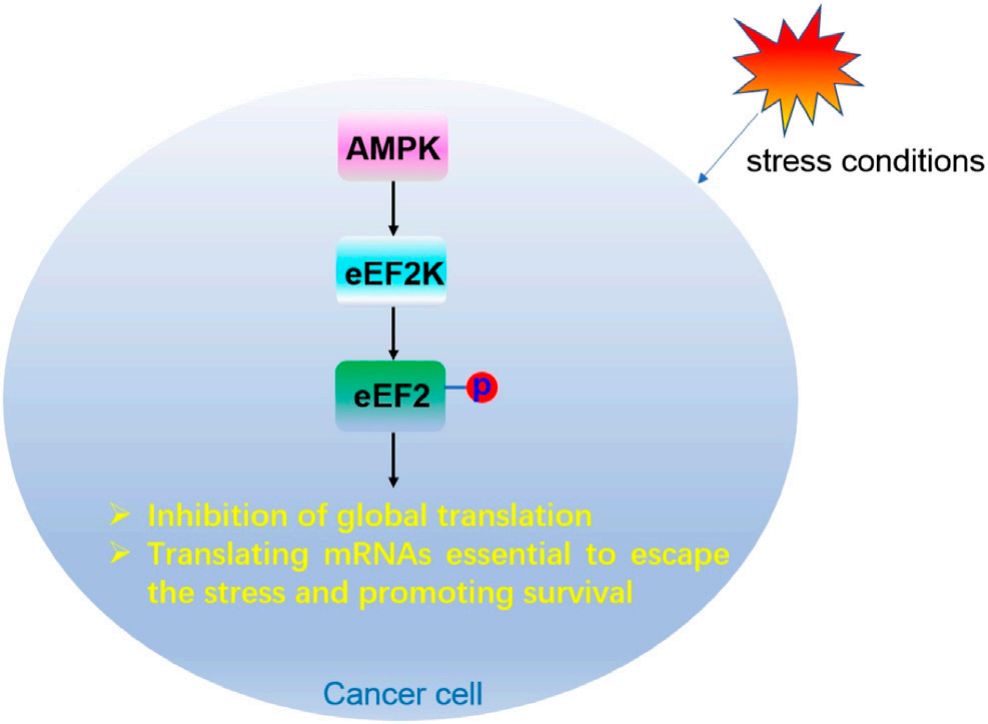
Regulation mechanism of eEF2 phosphorylation modification in cancer cells.

Under conditions of energy depletion or nutrient deprivation, tumor cells redistribute energy resources through weakening overall translation, while translating specific mRNAs to cope with stresses and fight for survival. AMPK, a sensor of energy, exists in a variety of tumors. When AMPK is activated, it can stimulate eEF2K, which further inhibits eEF2 activity by phosphorylating the Thr56 of eEF2. Inhibition of eEF2 leads to slowing down of protein translation (Proud, 2015), and prevents tumor cells from growing under nutritional deficiency (Leprivier et al., 2013) (Figure 8). Therefore, eEF2K is a negative regulator of protein synthesis (Wang et al., 2017), and inhibition of eEF2K activity may have therapeutic significance in preventing the survival of tumor cells and recovering protein translation.

eEF2K is an atypical kinase, and plays an important role in the migration and survival of tumor cells. It is overexpressed in glioblastoma (Leprivier et al., 2013), medulloblastoma, mammary cancer (Liu et al., 2014) and pancreatic tumor (Ashour et al., 2014). eEF2K is activated by stresses such as nutrition deficiency, hypoxia (Moore et al., 2015), acidosis (Xie et al., 2015) and cellular energy depletion (Browne et al., 2004). Inhibiting eEF2K decreases the invasion and migration of tumor cells, while deletion of eEF2 or eEF2K overexpression promotes wound healing and invasion. These results indicated that eEF2K has a protective function in tumor cells and therefore can be used as a molecular target to prevent cancer metastasis (Xie et al., 2018). Nevertheless, a recent study showed that eEF2K can protect cells under stress conditions and make tumor cells adapt to stresses (Leprivier et al., 2013). Therefore, stimulating eEF2K-eEF2 axis to suppress tumor requires serious assessment, and more in-depth studies are required to comprehensively evaluate its effectiveness from a treatment perspective (Knight et al., 2020).

### eIF5A and Tumorigenesis

**FIGURE 9:**
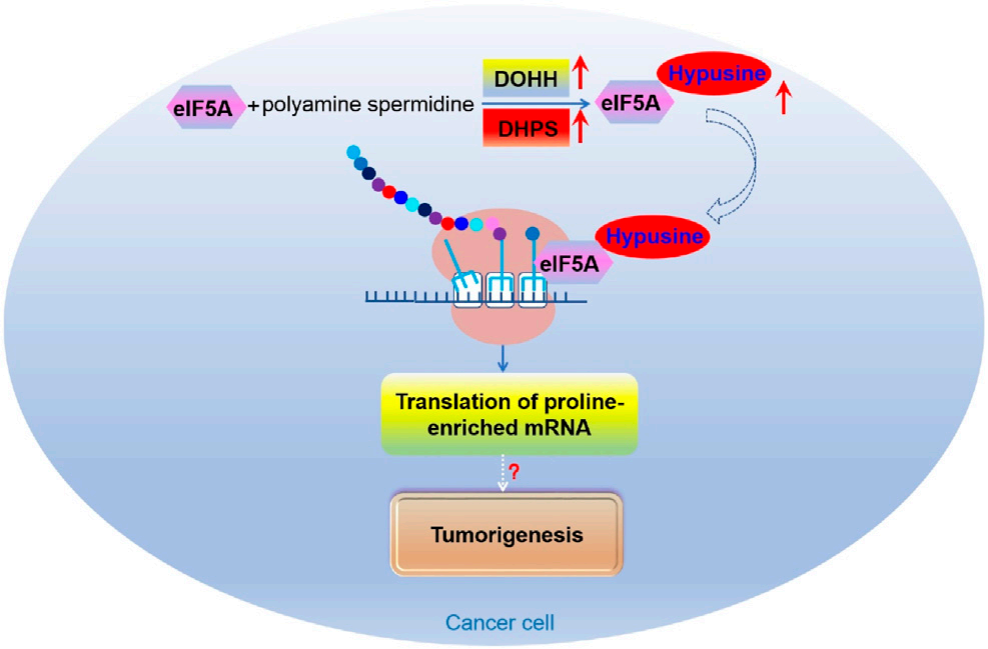
The role of eIF5A mediated translational control in tumorigenesis.

eIF5A is involved in the invasive and/or metastatic processes of several types of human cancer (Mathews and Hershey, 2015). There are two subtypes of eIF5A in mammals, both of which were modified with hypusine on the same lysine (Clement et al., 2003). eIF5A1 is widely distributed in rapidly proliferating cells while eIF5A2 is expressed in a tissue-specific manner and is almost undetectable in most cases (Clement et al., 2006). It was found that both eIF5A1 and eIF5A2 are associated with several malignancies (Mathews and Hershey, 2015). Interestingly, the expression of eIF5A1 and eIF5A2, along with DOHH and DHPS, are increased in multiple tumors (Nakanishi and Cleveland, 2016). Recently, it was found that eIF5A2 promotes doxorubicin resistance of colon cancer cells by regulating EMT, suggesting that inhibition of eIF5A2 can be used as a way to reverse the drug resistance of colorectal cancer (Figure 9) (Bao et al., 2015). An early study suggested that eIF5A2 may aid tumorigenesis via promoting the translation of some mRNAs that boost DNA replication and provoke excessive proliferation of tumor cells (Hanauske-Abel et al., 1995). Nevertheless, more in-depth studies on the function and molecular mechanism of eIF5A and its hypusination modification in tumors are needed.

## Concluding Remarks

The elongation phase of translation is an important regulatory node in health and disease. Dysregulation of this process is often related to various disorders including tumors, neurodegenerative diseases and cardiovascular diseases. The close link between translational factors and human diseases are well coincident with the concept that gene expression is accurately regulated at the translational level (Dever et al., 2018). There are multiple levels at which protein translation can be regulated to hinder disease progression. Clarifying how post-translational modifications control the activity of translational factors in tumors is helpful to reveal the regulation mechanism in tumorigenesis, and provides a rationale for the new interventional treatment (Xu and Ruggero, 2020). There are still many questions about translation elongation that need to be resolved: 1) The exact physiological function of phosphorylated EF-G and EF-Tu; 2) The molecular mechanism of how ADP-ribosylation impairs the function of eEF2; 3) The molecular mechanism of EF4 in protein translation under stress conditions; 4) The unique requirement of eEF3 in fungal and its action mechanism in translation elongation; 5) The feasibility of eEF2K as a tumor therapeutic target; 6) The molecular mechanism of eIF5A and its hypusine modification in protein translation and tumorigenesis.
